# Changes in Sexual Behaviors among HIV-Infected Individuals after Their HIV Diagnosis in a Rural Prefecture of Eastern China

**DOI:** 10.1371/journal.pone.0059575

**Published:** 2013-03-18

**Authors:** Haijiang Lin, Yingying Ding, Xing Liu, Weiming Zhu, Meiyang Gao, Na He

**Affiliations:** 1 Department of Epidemiology, School of Public Health, Fudan University, Shanghai, China; 2 Taizhou City Center for Disease Control and Prevention, Taizhou City of Zhejiang Province, China; 3 Department of Epidemiology, School of Public Health, University of California Los Angeles, Los Angeles, California, United States of America; 4 The Key Laboratory of Public Health Safety of Ministry of Education, Shanghai, China; Alberta Provincial Laboratory for Public Health/University of Alberta, Canada

## Abstract

**Objective:**

To describe changes in sexual behaviors among HIV-infected individuals after their HIV diagnosis.

**Methods:**

All HIV-infected individuals diagnosed by the end of 2009 in Taizhou Prefecture were invited to participate in this 12-month prospective study. Assessments including the total number and types of sexual contacts, and condom use details for up to their most familiar eight sexual contacts were collected both at enrollment and 12-month follow-up.

**Results:**

262 HIV-infected individuals were eligible for analysis. The total number of sexual contacts reported by participants was 4,017, 1,496 and 356 during the 12- month period prior to HIV diagnosis (T1), the 12-month period prior to the baseline survey (T2), and the 12-month follow-up period (T3), respectively. The difference in the number of sexual contacts between T2 and T1 was −5 in median (IQR −1, −14), and the difference between T3 and T2 was 0 in median (IQR: 0, −6). A larger proportion of spousal or long-term heterosexual contact was reported from T1(27.7%) to T2(42.5%) to T3(76.1%), whereas a smaller proportion of commercial heterosexual contacts was reported from T1 (48.6%) to T2 (33.2%) to T3 (7.0%) as well as a smaller proportion of non-commercial casual homosexual contacts was reported from T2 (8.4%) to T3 (3.8%).The proportion of consistent condom use increased significantly from T1 (9.3%) to T2 (35.3%) to T3 (91.5%).

**Conclusion:**

Sexual behaviors did not change in a uniform manner for the participants in our study. Sexual behaviors and sexual networks vis-à-vis HIV diagnosis and follow-up were associated with the participant’s characteristics and HIV infection and treatment status. The overall lesson is that individuals who are unaware of their HIV infection are the main drivers of secondary transmission. Early identification of HIV infection and access to antiretroviral therapy (ART ) are both key strategies to the control and prevention.

## Introduction

People who are unaware of their infection are unable to benefit from prevention and health care services and are at higher risk for transmitting human immunodeficiency virus (HIV) to others (i.e., secondary transmission) [1]–[4]. Therefore, identifying persons with undiagnosed HIV infection and linking them to prevention services continues to be a priority for HIV prevention and control [2]–[8]. The risk for HIV secondary transmission is dependent on the behaviors of the infected individual [9]–[11]. The changes of risky sexual behaviors mainly involve decrease in the number of sexual contacts, change of types of risky sexual contacts and increase in condom use. The international literature (e.g., United States, Zambia, Kenya, Tanzania, Trinidad, Puerto Rico, and India) reveals that people who are aware of their HIV- positive status tend to reduce their risk behaviors, and to adopt safer sex practices [12]–[19]. These may involve decrease in the number of sexual contacts and increase in condom use. Lessons learned from these “changes in behaviors” are critical for informing how to develop effective prevention and/or intervention strategies [10], [20], [21]. Nonetheless, changes in risky sexual behaviors of the independent HIV-infected individuals and risky behavioral network systems of interacting individuals among the HIV-infected individuals and their sexual contacts have been asserted to affect HIV transmission in the area, where sexual transmission is the most common route [11], [20]–[22]. In addition, in studying HIV transmission dynamics, substantial attention has traditionally been devoted to observations about risky sexual behaviors of the individual. However, these studies reveal that not only HIV related risky behavior plays a key role in the HIV transmission, but HIV related behavioral changes and patterns are the particularly important determinants of HIV epidemic at the population-level over time [9], [23], [24], and dynamic changes in the pattern of connections between HIV-infected individuals and their sexual contacts can often affect infection levels much more [23]–[26].

China has a fast growing HIV epidemic mostly due to sexual transmission, especially among high-risk groups such as men who have sex with men (MSM) and heterosexual men engaging in commercial sex [27]–[28]. In particular, there is a knowledge gap regarding how and in what way sexual network characteristics contribute to HIV transmission, especially secondary transmission. Therefore, to fill this gap, we designed and conducted a longitudinal study among a cohort of HIV-infected individuals in a prefecture of Eastern China to retrospectively examine their risky sexual behaviors and egocentric sexual networks in the past 12 months before HIV diagnosis and prospectively examine changes of risky sexual behaviors and egocentric sexual networks during a one year follow-up period after HIV diagnosis. Knowledge gained from this study will be valuable for understanding the HIV dynamic in China and for designing effective prevention and intervention programs targeting Chinese HIV/AIDS patients.

## Materials and Methods

### Ethics Statement

This study was approved by the Institutional Review Board (IRB) of Fudan University and written informed consent was obtained from all participants.

### Study Site and Determination of HIV Transmission Route

This study was conducted in Taizhou prefecture of Zhejiang Province, a coastal region in Eastern China, which has a total of 5.9 million residents(National Bureau of Statistics of China, unpublished data). By the end of 2011, 886 HIV/AIDS individuals had been diagnosed and registered with the Chinese National Information System for AIDS Prevention and Control (CNISAPC), with the majority (90%) diagnosed in the past five years. Among these reported HIV/AIDS individuals, 69.3% were infected through heterosexual transmission, 15.8% infected through homosexual transmission, and 7.3% infected through injection drug use (IDU). HIV transmission route was determined by the judgment of attending health professionals using three criteria. First, an HIV-infected individual was considered to be sexually transmitted only if s/he reported having never had any experience of IDU and blood/plasma donation or transfusion. Second, for an HIV-infected female, she must also reported having had unprotected sex with an HIV-positive spouse or having engaged in commercial or non-commercial extramarital sex in order to be considered as heterosexually transmitted. Third, for a HIV-infected male, he would be considered as homosexually transmitted if he reported having had unprotected oral and/or anal sex with another man. If he did not meet these criteria, he would be considered as heterosexually transmitted.

### Study Participants and Data Collection

All HIV-infected individuals who were diagnosed and registered with the Chinese National Information System for AIDS Prevention and Control (CNISAPC) by the end of 2009 in Taizhou prefecture and aged 18 years or above were invited and written informed consent to participate in a cohort study with a total of 12-month follow-up. The baseline and the follow-up survey were conducted at the end of 2009 and 2010, respectively. In both surveys, a face-to-face questionnaire interview was administered by a public health professional in private settings, mostly in HIV Voluntary Counseling and Testing (VCT) room in the local Center for Disease Control and Prevention (CDC). At the baseline survey, sexual information including the number of sexual contacts, and the type of sexual contacts and condom use with up to a maximum of eight most familiar sexual contacts during the 12 months period prior to HIV diagnosis (T1) and during the 12 months period prior to baseline survey (T2) were requested separately. At the follow-up survey, the above-mentioned sexual information during the 12-month follow-up period (T3) (i.e., 12 months period prior to the follow-up survey) was requested. It is of note that T1 and T2 overlapped to some extent for those diagnosed with HIV infection for less than 12 months at the baseline survey. In addition, participants’ sociodemographic characteristics, HIV transmission route and use of antiretroviral therapy (ART) were abstracted from the Chinese National Information System for AIDS Prevention and Control (CNISAPC). Each participant received 30 Yuan (about US$5) for travel reimbursement at each visit.

### Routine Intervention and Health Care Services for HIV-infected Individuals

According to the national guidelines, all diagnosed and registered HIV-infected individuals in China should receive routine prevention and health care services. These include but are not limited to post-diagnosis HIV counseling, partner notification and HIV testing, condom promotion, monitoring of disease progression and if necessary, free ART [29].

### Statistical Analyses

Data were analyzed using SPSS 11.5 (version 17.0) for Windows (SPSS Inc., Chicago, USA). Medians and interquartile ranges (IQR) of the number of sexual contacts of HIV-infected individuals at different time periods by socio-demographic characteristics, HIV transmission route and ART status were presented. Difference in the number of sexual contacts of HIV individuals among HIV-infected individuals between different time points was compared by Wilcoxon signed-rank test because none fulfilled the Kolmogorov-Smirnov test for normality. To determine whether number of sexual contacts prior to HIV diagnosis (T1) or the changes in the number of sexual contacts of HIV-infected individuals among HIV-infected individuals during different time periods were associated with socio-demographic characteristics, HIV transmission route and ART status, Mann-Whitney test (variables with two categories) and Kruskal-Wallis test (variables with three or more categories) were applied. Finally, Chi-squared test or Fisher’s exact was used to compare the proportion of types of sexual contacts or condom use of HIV individuals between different time points. Significance was determined at the *P*<0.05 level.

## Results

**Figure 1:**
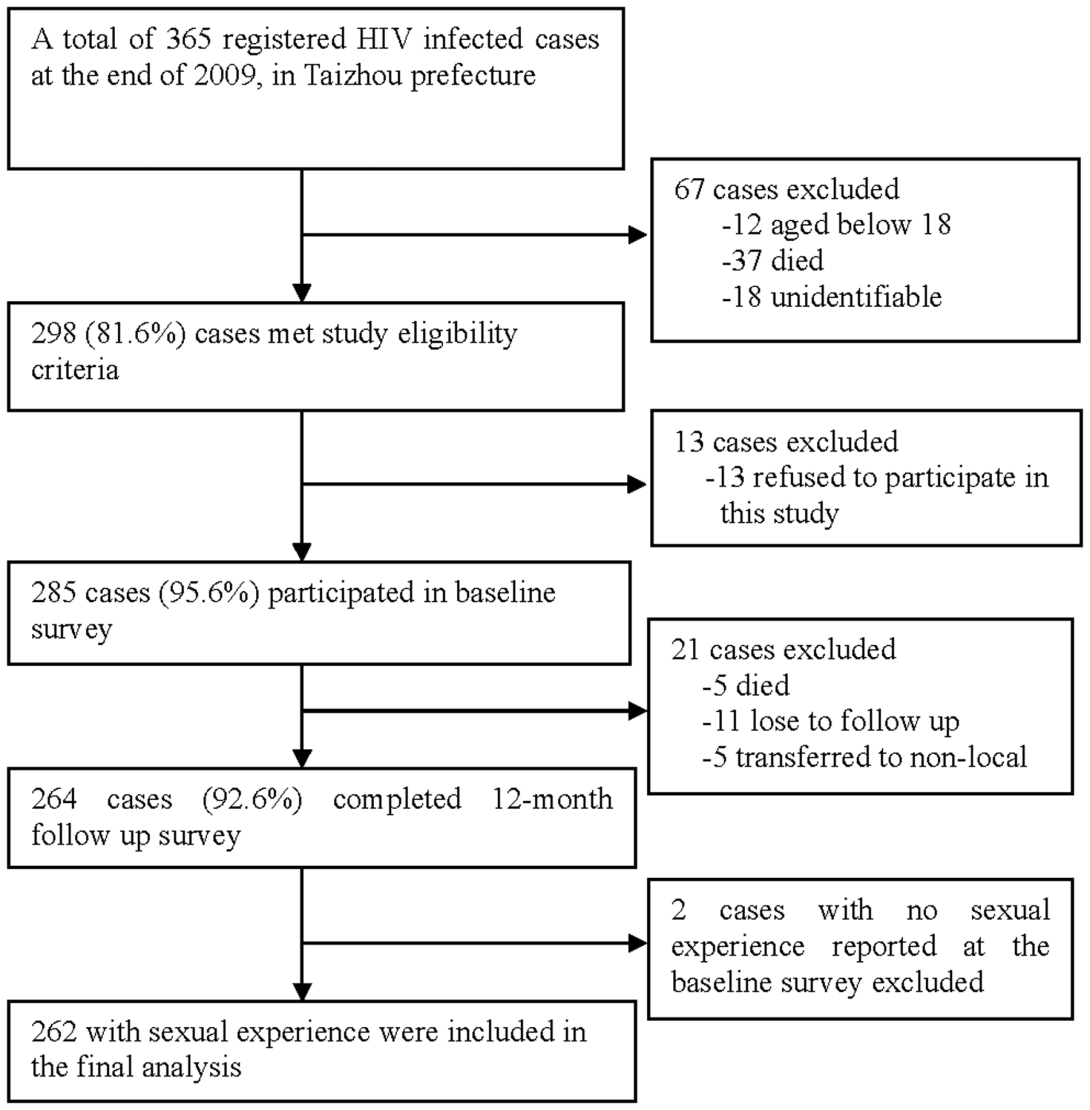
A schematic flow chart of the study sample.

Figure 1 depicts the flow of the study cohort. In brief, 298 (81.6%) out of the total of 365 registered HIV-infected individuals at the beginning of the study met the study criteria and were invited to participate in the study. Of them, 285 (95.6%) provided written informed consent to participate in the baseline survey. Among these baseline participants, 264 (92.6%) completed the follow-up survey. However, only the 262 (99.2%) HIV-infected individuals who had ever had sexual experience and participated in both the baseline and the follow-up surveys were included in the analysis.

### Socio-demographic and HIV Infection Characteristics

Among the 262 participants, 67.6% were male, 69.4% aged between 18 and 39 years, 73.7% were married, 38.5% were illiterate or received only primary school education, 81.3% were infected with HIV through heterosexual transmission and 12.6% were infected homosexually, and 72.9% received ART (Table 1). At the time of the baseline survey, 98 participants (37.5%) had been diagnosed with HIV infection for 24 months or more, 63 (24.0%) for at least 12 months but less than 24 months, 36 (13.7%) for at least 6 months but less than 12 months, and 65 (24.8%) for less than 6 months (Table 1).

**Table 1:** Sexual contacts of HIV individuals in the 12 months period prior to HIV diagnosis (T1), the baseline survey (T2) and the one-year follow-up survey (T3), respectively, in Taizhou prefecture, 2009–2010.

Characteristics	No. (%)	No. of sexual contacts at different time points Median (IQR)				No. (%) of HIV individuals with reduced sexual contacts between different time points		Difference of number of sexual contacts of HIV individuals between different time points Median (IQR)				
		T1	*P* value	T2	T3	T2-T1	T3-T2	T2-T1	*P* value		T3-T2 *P* value	
**Overall**	262 (100.0)	12 (5, 20)		2 (1, 8)	1 (0, 2)	221 (84.4)	119 (56.9)	−5 (−1, −14)		P*<0.01	0 (0, −6)	P*<0.01
**Gender**												
Male	177 (67.6)	13 (7, 24)	*P* † <0.01	3 (1, 8)	1 (0, 2)	163 (92.1)	90 (65.7)	−6 (−2. −14)		*P* † <0.01	−1 (0, −6)	*P* † <0.05
Female	85 (32.4)	7 (1, 15)		1 (1, 6)	1 (0, 2)	58 (68.2)	29 (40.3)	−2 (0, −7)			0 (0, −2)	
**Age group(yr)**												
18–29	91 (34.7)	13 (6, 22)	*P* ‡ >0.05	2 (1, 0)	1 (0, 2)	76 (83.5)	45 (57.0)	−5 (−1, −14)		*P* ‡ >0.05	0 (0, −7)	*P* ‡ <0.05
30–39	91 (34.7)	11 (5, 18)		1 (0, 4)	1 (0, 2)	75 (82.4)	29 (43.3)	−6 (−1, −4)			0 (0, −3)	
40–49	39 (14.9)	12 (6, 20)		3 (1, 10)	2 (0, 2)	34 (87.2)	22 (68.8)	−5 (−2, −16)			−1 (0, −8)	
50–68	41 (15.6)	10 (5, 15)		4 (1, 7)	2 (0, 2)	36 (87.8)	23 (74.2)	−2 (−1, −9)			−2 (0, −5)	
**Marital status**												
Currently married	193 (73.7)	9 (1, 21)	*P* ‡ <0.05	4 (1, 10)	1 (0, 2)	159 (82.4)	75 (47.8)	−5 (−1, −13)		*P* ‡ >0.05	0 (0, −6)	*P* ‡ <0.01
Never married	48 (18.3)	12 (5, 15)		1 (0, 6)	1 (0, 2)	43 (89.6)	32 (84.2)	−8 (−2, −15)			−3 (0, −7)	
Divorced or widowed	21 (8.0)	12 (6, 21)		2 (1, 8)	1 (0, 2)	19 (90.5)	12 (85.7)	−4 (−1, −15)			−2 (0, −4)	
**Education**												
Illiterate or elementary school	101 (38.5)	10 (5, 18)	*P* ‡ >0.05	1 (0, 7)	1 (0, 2)	88 (87.1)	44 (57.9)	−5 (−1, −12)		*P* ‡ >0.05	0 (0, −6)	*P* ‡ >0.05
Junior middle school	110 (42.0)	13 (6, 21)		1 (1, 6)	1 (0, 2)	92 (83.6)	47 (51.1)	−1 (−6, −16)			0 (0, −4)	
Senior middle school or above	51 (19.5)	12 (5, 15)		5 (1, 10)	1 (0, 2)	41 (80.4)	28 (68.3)	−4 (−1, −12)			−1 (0, −8)	
**Transmission route**												
Heterosexual	213 (81.3)	11 (5, 16)	*P* ‡ <0.01	1 (1, 6)	1 (0, 2)	181 (85.0)	89 (52.7)	−5 (−1, −11)		*P* ‡ <0.01	0 (0, −4)	*P* ‡ <0.01
Homosexual	33 (12.6)	36 (15, 40)		10 (2,25)	2 (0, 3)	29 (87.9)	24 (82.8)	−17 (−5, −30)			−7 (0, −23)	
Others	16 (6.1)	2 (1, 11)		1 (0, 6)	0 (0, 1)	11 (68.8)	6 (54.5)	−1 (0, −5)			0 (0, −1)	
**Official residence**												
Local	195 (74.4)	12 (6, 20)	*P* † <0.01	2 (1, 8)	1 (0, 2)	166 (85.1)	86 (52.8)	−5 (−2, −15)		*P* † >0.05	0 (0, −6)	*P* † >0.05
Non-local	67 (25.6)	12 (4, 18)		1 (0, 10)	0 (0, 2)	56 (83.6)	33 (71.7)	−5 (−1, −12)			0 (0, −7)	
**Received ART**												
Yes	191 (72.9)	13 (6, 24)	*P* † <0.01	2 (1, 8)	1 (0, 2)	157 (82.2)	79 (51.0)	−6 (−2, −16)		*P* † <0.01	0 (0, −6)	*P* † >0.05
No	71 (27.1)	8 (4, 14)		2 (1, 8)	1 (0, 2)	64 (90.1)	40 (74.1)	−3 (−1, −7)			0 (0, −6)	
**Time between HIV diagnosis and baseline survey (months)**												
<6	65 (24.8)	14 (8, 2)	*P* ‡ <0.01	10 (6, 20)	2 (0, 2)	49 (75.4)	63 (96.9)	−2 (0, −6)		*P* ‡ <0.01	−8 (−4, −18)	*P* ‡ <0.01
6–	36 (13.7)	11 (6, 21)		8 (5, 16)	2 (0, 2)	30 (83.3)	33 (91.7)	−2 (−1, −6)			−7 (−3, −10)	
12–	63 (24.0)	14 (8, 23)		1 (1, 3)	1 (0, 2)	58 (92.1)	19 (38.0)	−12 (−5, −21)			0 (0, −2)	
24–	98 (37.5)	7 (1, 16)		1 (0, 1)	1 (0, 1)	84 (85.7)	4 (6.9)	−6 (−1, −14)			0 (0, 0)	

**Note:** *Wilcoxon signed-rank test compare the difference in number of sexual contacts of HIV individuals between different time points;

† Mann-Whitney test for variables with two categories were applied to determine whether the number of sexual contacts during T1 or the changes in the number of sexual contacts of HIV-infected individuals during different time periods were significantly associated with their sociodemographic, HIV transmission route and ART status;

‡ Kruskal-Wallis test for variables with three or more categories were applied to determine whether were significantly associated with their sociodemographic, HIV transmission route and ART status.

### Sexual Contacts during the 12-month Period Prior to HIV Diagnosis (T1)

#### The total number of all reported sexual contacts during this period

262 sexually experienced participants reported a total of 4,017 sexual contacts during the 12-month period prior to their HIV diagnosis (T1). As shown in Table 1, the median number of reported sexual contacts was 12 (IQR: 5, 20). Homosexually infected participants reported a significant higher number of sexual contacts (median: 36, IQR: 15, 40) than those infected heterosexually (median: 11, IQR: 5, 16) (*P*<0.01) (Table 1).

#### The number of most familiar sexual contacts during this period

During the 12 months period prior to their HIV diagnosis (T1), the 262 sexually experienced participants reported a total of 871 most familiar sexual contacts (Table 2). The average number of most familiar sexual contacts per participant was 3.3 (871/262) overall, 3.1 (661/213) for those infected heterosexually, 5.4 (177/33) for those infected homosexually, and 2.1 (33/16) for those infected via other route(s) (Table 2).

**Table 2:** Sexual relationship and condom use of HIV individuals with their most familiar sexual contacts in the 12 months period prior to HIV diagnosis (T1), the baseline survey (T2) and the one-year follow-up survey (T3), respectively, in Taizhou prefecture, 2009–2010.

	No. of most familiar sexual contracts reported by heterosexually infected HIV individuals						No. of most familiar sexual contracts reported by homosexually infected HIV individuals						No. of most familiar sexual contracts reported by otherwise infected HIV individuals						No. of most familiar sexual contracts reported by all HIV individuals					
	(N1 = 213) at different time points						(N2 = 33)at different time points						(N3 = 16) at different time points						(N = 262) at different time points					
	T1		T2		T3		T1		T2		T3		T1		T2		T3		T1		T2		T3	
	(n = 661, %)		(n = 303, %)		(n = 156, %)		(n = 177, %)		(n = 104, %)		(n = 45, %)		(n = 33, %)		(n = 21, %)		(n = 12, %)		(n = 871, %)		(n = 428, %)		(n = 213, %)	
Type of sexual contacts			*P* † <0.01		*P* ‡<0.01				*P* † >0.05		*P* ‡<0.05				*P* † <0.05		*P* ‡>0.05				*P* † <0.01		*P* ‡<0.01	
Spousal or long-term heterosexual contacts	204	30.9	153	50.5	135	86.5	21	11.9	19	18.3	20	44.4	16	48.5	10	47.6	7	58.3	241	27.7	182	42.5	162	76.1
Commercial heterosexual contacts	380	57.5	124	40.9	11	7.1	30	16.9	15	14.4	4	8.9	13	39.4	3	14.3	–	–	423	48.6	142	33.2	15	7
Non-commercial casual heterosexual contacts	77	11.6	26	8.6	10	6.4	9	5.1	5	4.8	–	–	4	12.1	8	38.1	5	41.7	90	10.3	39	9.1	15	7
Long-term homosexual contacts	–	–	–	–	–	–	16	9	8	7.7	2	4.4	–	–	–	–	–	–	16	1.8	8	1.9	2	0.9
Commercial homosexual contacts	–	–	–	–	–	–	34	19.2	21	20.2	11	24.4	–	–	–	–	–	–	34	3.9	21	4.9	11	5.2
Non-commercial casual homosexual contacts	–	–	–	–	–	–	67	37.8	36	34.6	8	17.8	–	–	–	–	–	–	67	7.7	36	8.4	8	3.8
**Condom use of HIV individuals with their sexual contacts**			***P*** † **<0.01**		***P*** ‡ **<0.01**				***P*** † **<0.01**		***P*** ‡ **<0.01**				***P*** † **<0.01**		***P*** ‡ **<0.01**				***P*** † **<0.01**		***P*** ‡ **<0.01**	
Consistent	67	10.1	116	38.3	145	92.9	11	6.2	25	24	38	84.4	3	9.1	10	47.6	12	100	81	9.3	151	35.3	195	91.5
Inconsistent	287	43.4	76	25.1	11	7.1	80	45.2	32	30.8	7	15.6	9	27.3	3	14.3	–	–	376	43.2	111	25.9	18	8.5
Never	307	46.4	111	36.6	–	–	86	48.6	47	45.2	–	–	21	63.6	8	38.1	–	–	414	47.5	166	38.8	–	–

**Note:** n refers to the total number of sexual contacts reported;

† Chi-square test or Fisher’s exact test compare the proportion of types of sexual contacts or condom use of HIV individuals between T2 and T1;

‡ Chi-square test or Fisher’s exact test compare the proportion of types of sexual contacts or condom use of HIV individuals between T3 and T2.

#### Sexual relationship and condom use of participants with their most familiar sexual contacts during this period

Of the total of 661 most familiar sexual contacts reported by heterosexually infected participants, 204 (30.9%) were spousal or long-term heterosexual contacts, 380 (57.5%) were commercial heterosexual contacts and 77 (11.6%) were non-commercial casual heterosexual contacts. Condoms were consistently used for only 67 (10.1%) sexual contacts, whereas condoms were never used for 307 (46.4%) sexual contacts (Table 2). Of the total of 177 most familiar sexual contacts reported by homosexually infected participants, 21 (11.9%) were spousal or long-term heterosexual contacts, 30 (16.9%) were commercial heterosexual contacts, 16 (9.0%) were long-term homosexual contacts, 34 (19.2%) were commercial homosexual contacts and 67 (37.8%) were non-commercial casual homosexual contacts.Condoms were consistently used in only 11 (6.2%) sexual, whereas condoms were never used in 86 (48.6%) sexual contacts (Table 2).

### Sexual Contacts during the 12-month Period Prior to the Baseline Survey (T2)

#### The total number of all reported sexual contacts during this period

At the baseline survey (T2), 262 participants reported a total of 1,496 sexual contacts in the past 12 months, with 56 (21.4%) reported no sex in this period. As shown in Table 1, the median number of reported sexual contacts was 2 (IQR: 1, 8). Homosexual infected participants reported a higher number of sexual contacts (median: 10, IQR: 2, 25) than those infected heterosexually (median: 1, IQR: 1, 6) (Table 1).

#### The number of most familiar sexual contacts during this period

During the 12 months period prior to the baseline survey (T2), the 262 participants reported a total of 428 most familiar sexual contacts (Table 2). The average number of most familiar sexual contacts per participant was 1.6 (428/262) overall, 1.4 (303/213) for those infected heterosexually, 3.1 (104/33) for those infected homosexually, and 1.3 (21/16) for those infected via other routes (Table 2).

#### Sexual relationship and condom use of participants with their most familiar sexual contacts during this period

Among the total of 303 most familiar sexual contacts reported by heterosexually infected participants, 153 (50.5%) were spousal or long-term heterosexual contacts, 124 (40.9%) were commercial heterosexual contacts and 26 (8.6%) were non-commercial casual heterosexual contacts. Condoms were consistently used in 116 (38.3%) sexual contacts, whereas condoms were never used in 111 (36.6%) sexual contacts. Among the total of 104 most familiar sexual contacts reported by homosexually infected participants, 19 (18.3%) were spouse or long-term heterosexual contacts, 15 (14.4%) were commercial heterosexual contacts, 8 (7.7%) were long-term homosexual contacts, 21 (20.2%) were commercial homosexual contacts and 36 (34.6%) were non-commercial casual homosexual contacts, and condoms were consistently used for 25 (24.0%) sexual contacts whereas condoms were never used for 47 (45.2%) sexual contacts.

### Sexual Contacts during the 12 months Period Prior to the Follow-up Survey (T3)

#### The total number of all reported sexual contacts during the follow-up period

At the follow-up survey (T3), the 262 participants reported a total of 356 sexual contacts in the past 12 months, i.e., during the 12-month follow-up period of the study, with 93 (35.5%) reported no sex in this period. As shown in Table 1, the median number of reported sexual contacts was 1 (IQR: 0, 2) overall, 2 (IQR: 0, 3) for homosexually infected participants and 1 (IQR: 0, 2) for those infected heterosexually (Table 1).

#### The number of most familiar sexual contacts during the follow-up period

During the 12-month follow-up period, the 262 participants reported a total of 213 most familiar sexual contacts (Table 2). The average number of most familiar sexual contacts per participant was 0.8 (213/262) overall, 0.7 (156/213) for those infected heterosexually, 1.4 (45/33) for those infected homosexually, and 0.7 (12/16) for those infected otherwise (Table 2).

#### Sexual relationship and condom use of participants with their most familiar sexual contacts during the follow-up period

Among the total of 156 most familiar sexual contacts reported by heterosexually infected participants, 135 (86.5%) were spouse or long-term heterosexual contacts, 11 (7.1%) were commercial heterosexual contacts and 10 (6.4%) were non-commercial casual heterosexual contacts. Condoms were consistently used in145 (92.9%) sexual contacts, whereas none of them reported never condom use. Among the total of 45 most familiar sexual contacts reported by homosexually infected participants, 20 (44.4%) were spouse or long-term heterosexual contacts, 4 (8.9%) were commercial heterosexual contacts, 2 (4.4%) were long-term homosexual contacts, 11 (24.4%) were commercial homosexual contacts and 8 (17.8%) were non-commercial casual homosexual contacts, and condoms were consistently used in 38 (84.4%) sexual contacts whereas none of them reported never condom use.

### Changing Sexual Behaviors from the Time of HIV Diagnosis (T1) to the Baseline Survey (T2)

#### The total number of all reported sexual contacts in the past 12 months

Compared with the time of HIV diagnosis, 221 (84.4%) of the participants reported less numbers of sexual contacts in the past 12 months at the baseline survey (Table 1). This difference in the number of sexual contacts in the past 12 months reported by the participants at the baseline survey versus at the time of HIV diagnosis was −5 in median (IQR: −1, −14) and was statistically significant. Such a difference was also statistically significant by participants’ characteristics including gender, HIV transmission route, status of ART and the time length from HIV diagnosis to the baseline survey (Table 1). Participants who were male, homosexually infected with HIV, receiving ART and had a longer time from HIV diagnosis to the baseline survey had more reductions in the number of sexual contacts in the past 12 months.

#### Sexual relationship and condom use of participants with their most familiar sexual contacts in the past 12 months

As shown in Table 2, the participants reported a less number of most familiar sexual contacts in the past 12 months at the baseline survey versus at the time of HIV diagnosis (428 vs. 871). Notably, the type of most familiar sexual contacts was significantly different between the baseline survey and the time of HIV diagnosis. Compared with the time of HIV diagnosis, the participants especially those heterosexually infected with HIV reported a larger proportion of spousal or long-term heterosexual contact but a smaller proportion of commercial heterosexual contacts at the baseline survey (Table 2). A significantly higher proportion of consistent condom use (35.3%) was also reported by participants at the baseline survey (Table 2).

### Changing Sexual Behaviors from the Baseline Survey (T2) to the Follow-up Survey (T3)

#### The total number of all reported sexual contacts in the past 12 months

Compared with the baseline survey, 119 (56.3%) of the participants reported less numbers of sexual contacts in the past 12 months at the follow-up survey (Table 1). This difference in the number of sexual contacts in the past 12 months reported by the participants at the follow-up survey versus at the baseline survey was 0 in median (IQR: 0, −6) and was statistically significant. Such a difference was also statistically significant by participants’ characteristics including gender, age, marital status, HIV transmission route and the time length from HIV diagnosis to the baseline survey (Table 1). Participants who were male, elder, never married, homosexually infected with HIV, receiving ART and had a longer time from HIV diagnosis to the baseline survey had more reductions in the number of sexual contacts in the 12 months.

#### Sexual relationship and condom use of participants with their most familiar sexual contacts in the past 12 months

As shown in Table 2, the participants reported a less number of most familiar sexual contacts in the past 12 months at the follow-up survey versus at the baseline survey (213 vs. 428). Notably, the type of most familiar sexual contacts was significantly different between the follow-up survey and the baseline survey. Compared with the baseline survey, the participants reported a larger proportion of spouse or long-term heterosexual contact but a smaller proportion of commercial heterosexual contacts and non-commercial casual homosexual contacts at the follow-up survey (Table 2). A very high proportion (91.5%) of consistent condom use was also reported by participants at the follow-up (Table 2).

## Discussion

This study, for the first time, examined the longitudinal changes in sexual behaviors among a diverse cohort of HIV–infected individuals in China. However, sexual behaviors did not change in a uniform manner for the participants in the study. That is, sexual behaviors and sexual networks vis-à-vis HIV diagnosis and follow-up were associated with the participant’s characteristics and HIV infection and treatment status. The significant association between reduction in the number of sexual contacts after HIV diagnosis and the time length from HIV diagnosis to the survey further underscores the importance of early identification of HIV infection status in prevention of HIV transmission. Male HIV individuals had more reductions in the number of sexual contacts than female individuals. This could be due to the fact that male individuals generally had more sexual contacts than female individuals and thus had more space and opportunities to reduce sexual contacts. More specifically, homosexually infected individuals in the study reported much more sexual contacts than other individuals when they were undiagnosed, i.e., unaware of HIV infection status. Studies in China have documented that men who have sex with men (MSM) are generally engaging in multiple sexual partnerships and most complicated sexual networks and are thus at highest risk of HIV infection in China [30]–[36]. Fortunately, the present study indicated that homosexually infected HIV individuals significantly reduced the number of sexual contacts after HIV diagnosis and over the follow-up by receiving routine intervention and health care services.

Although we do not have measures to ascertain people’s motivations for their changes in sexual behaviors over time, however, it is safe to assume that once people know their HIV- positive status they are likely to adjust and modify their lifestyles. This supposition is consistent with Halperin et al observation that behavioral changes mainly involved the reductions in extramarital, commercial, and casual sexual relations, and were followed by associated reductions in contact concurrency associated with HIV reduction [37].

The use of ART has been associated with a significant reduction in infectivity by effective viral suppression and therefore diminished risk of HIV transmission in many countries [38]–[45]. Nevertheless, the benefit of ART in preventing new HIV infections could also be through the mechanism that by regularly attending clinics prescribing ART, HIV patients receiving ART might have more opportunities to receive counseling and behavioral interventions and are therefore more likely to reduce risky behaviors than ART-naïve patients, as indicated by the present study that participants under ART did have more reductions in the number of sexual contacts than ART-naïve participants. From this perspective, China may benefit more than expected from the most recent initiatives of scaling up ART to patients at early disease stages and to highly risky groups such as MSM and female sex workers [46].

The overall lesson is that individuals who are unaware of their HIV infection are the main drivers of secondary transmission. Thus, early identification of HIV infection and access to ART are both key strategies to the control and prevention.

Our findings are timely and important as China is expanding and intensifying its anti-HIV campaigns especially HIV testing and surveillance, behavioral and biomedical interventions for HIV-infected individuals including the treatment as prevention (TasP) strategy in the coming years[4], [29], [47]–[48].

Nonetheless, this study has several limitations. First, this study was conducted in a prefecture area in Eastern China where the HIV epidemic is spreading and predominated with sexual transmission, which may be neither representative nor generalizable. Second, sexual behaviors were self-reported and therefore subject to recall bias, especially for those diagnosed with HIV infection two or more years ago; however, the proportion of these subjects was small and might not affect the results. Also, participants might over-report reductions in the number of sexual contacts over HIV diagnosis and the follow-up due to social desirability. To minimize these problems, all interviews were performed in private and patient-friendly places mostly in VCT sites and were administered by well-trained and experienced health professionals. Participants were encouraged, without judgments and blames to the victims, to provide information as real as possible. Another source of information bias was due to overlap between T1 and T2 among those diagnosed with HIV infection for less than 12 months, sexual behaviors before HIV diagnosis may be partly included in T2, which may lead to underestimation of the reduction in risky sexual behaviors from T1 to T2; nevertheless, we observed significant reduction in number of sexual contacts as well as larger proportion of spouse or long-term heterosexual contact and smaller proportion of commercial heterosexual contacts from T1 to T2. In addition, because of the relatively short follow-up, it cannot be determined whether the reductions in risky sexual behaviors and risky sexual networks among HIV-infected cases are sustainable over the longer term.

In summary, sexual behaviors and sexual networks of HIV infected individuals vis-à-vis HIV diagnosis and follow-up in the study changed significantly, and were associated with the participant’s characteristics and HIV infection and treatment status. The overall lesson is that individuals who are unaware of their HIV infection are the main drivers of secondary transmission. Early identification of HIV infection and access to ART are both key strategies to the control and prevention of HIV transmission.
